# SARS-CoV-2 outbreaks in long-term care facilities during the Omicron era in Québec, Canada

**DOI:** 10.1038/s41598-025-32967-y

**Published:** 2025-12-23

**Authors:** Radhouene Doggui, Manale Ouakki, Annick Boulais, Geneviève Deceuninck, Rodica Gilca, Étienne Racine, Christine Lacroix, Élise Fortin

**Affiliations:** 1 Direction des Risques Biologiques, Institut national de santé publique du Québec, Québec, QC Canada; 2 Axe Maladies infectieuses et immunitaires, Centre de Recherche du Centre Hospitalier Universitaire de Québec-Université Laval, Québec, QC Canada; 3 Département de Médecine Sociale et Préventive, Faculté de Médecine, Université Laval, Québec, QC Canada; 4 Département des Sciences de la santé Communautaire, Faculté de Médecine et des Sciences de la santé Communautaire, Université de Sherbrooke, Sherbrooke, QC Canada; 5 Département de Microbiologie, Infectiologie et Immunologie, Faculté de Médecine, Université de Montréal, Montréal, QC Canada

**Keywords:** COVID-19, Infection prevention and control, Outbreak, Canada, Nursing home, Viral infection, Population screening, Public health, Epidemiology

## Abstract

Residents of long-term care facilities (LTCFs) are at a high risk of severe COVID-19. Our study analyzed the COVID-19 outbreaks, associated cases and case fatality ratio (CFR) in LTCFs from May-2022 to September-2024 in Québec, Canada. An ecological analysis was conducted including all active LTCFs during the study period (*n* = 471). Regression analyses using generalized estimating equations were used to model odds of an active COVID-19 outbreak, confirmed COVID-19 cases incidence rate ratio (IRR) and ratio of CFR over different periods (wave 7, 2022-23 season, 2023-24 season), regions and LTCF bed capacity. A total of 2,501 outbreaks were recorded, corresponding to 39,089 COVID-19 cases (among residents and health care workers). The odds of an active outbreak, IRR of COVID-19 cases, and ratio of CFR declined significantly during the 2023-24 season compared to wave 7 (-35%, -39% and -24%, respectively; *p-values* < 0.0001). While larger LTCFs (≥ 40 beds) were up to 7 times more likely to have an active outbreak vs.10–39 beds, COVID-19 IRR and ratio of CFR were comparable across LTCFs’ bed-capacity. In summary, the burden of COVID-19 on LTCF residents has declined from May 2022 to September 2024. Bed capacity seems to predict outbreak occurrence but not virus transmission within LTCFs.

**Supplementary Information:**

The online version contains supplementary material available at 10.1038/s41598-025-32967-y.

## Introduction

As of November 24th, 2024, the World Health Organization has estimated that approximately 777 million cases and 7 million deaths to COVID-19 occurred globally since the beginning of the pandemic^1^. It is well established that older adults are particularly vulnerable to severe COVID-19 illness and death^2–4^. Residents of long-term care facilities (LTCFs) are at even higher risk because of their frailty and the presence of multiple chronic health conditions^5^. Close living quarters and communal environments inherent to LTCFs facilitate the rapid spread of infectious diseases, making LTCFs hotspots for respiratory infection outbreaks^3^. A meta-analysis conducted before the widespread use of COVID-19 vaccines showed that the hospitalization and case fatality proportions for SARS-CoV-2 laboratory-confirmed infection among aged care facilities were 37% and 23%, respectively^6^. An international study (across 22 high-income countries) estimated that the number of care home resident COVID-19 deaths represented 41% of all COVID-19 deaths in 2020–2021^7^. In Canada, from the beginning of the pandemic until July 24th, 2021, the proportion of LTCF residents who died from COVID-19 was 1,640 per 100,000 (number of LTCF beds were used as proxy for number of residents), corresponding to a COVID-19 mortality rate of about 1,100 per 100,000 person-years and to 73.3% of all deaths among elderly (≥ 65 years)^8^. In the province of Québec, an early report showed that 64% of total COVID-19 deaths occurred in LCTF residents by late November 2020^9^.

Several measures have been implemented in Québec to mitigate SARS-CoV-2 spread in LTCFs, including visitation restrictions, reduced crowding, social distancing, caregiver training, and vaccination^10–12^. Vaccination has drastically reduced the burden of COVID-19 disease among both residents and healthcare workers (HCWs)^13,14^. For instance, in Québec, the incidence rate of COVID-19 cases and deaths in LTCFs decreased by 92% and 95%, respectively, between February and April 2021, following the administration of the first vaccine dose^15^.

However, despite high vaccination coverages and a lower virulence of the virus, highly transmissible Omicron variants (such as BA.1 and BA.5)^16,17^ continued to cause outbreak-associated morbidity and mortality in LTCFs in different countries^16,18^. According to the European Centre for Disease Prevention and Control, based on data from seven countries, the average case-fatality ratios (CFR) declined from 21.3% during the fourth quarter of 2020 to 3–4% throughout 2022 and until the first quarter of 2023^16^. This is consistent with the lower risk of severe COVID-19 illness due to Omicron variants compared to pre-Omicron variants in the general population^19^. For example, a large Canadian retrospective study among patients presenting for bloodwork showed a decrease of CFR from mid-July to December 2022 among those aged 70–79 years (0.11%) and ≥ 80 years (1.27%), compared to the period marked by the co-circulation of the Delta and Omicron BA.1 variants (0.97% for 70–79 years and 3.32% for ≥ 80 years)^20^.

The present study analyzed the outbreaks in LTCFs from May 2022 (epiweek 22) to September 2024 (epiweek 38) in Québec (Canada). The main goal of this ecological study was to estimate the odds of COVID-19 outbreak on any given week, the incidence rate of COVID-19 and COVID-19 CFR in LTCFs in Québec, Canada over different periods, regions and LTCF bed capacity. This study is informative regarding the fact that the last assessment was conducted in Québec before the Omicron surge. Furthermore, to our knowledge, no studies have been conducted during the same surveillance period, or overlapping with it, that have examined the association between LTCF characteristics, and the incidence of COVID-19 outbreak associated morbidity.

## Methods

### Study design and population

About 9.3% of the Québec population is aged 75 and over^21^. In 2021, in Québec, there were 54 LTCF beds per 1000 persons aged 75 years or more^22^. The average LTCF length of stay has been estimated at 823 days (2.3 years) in 2020-21^22^. Our study includes all LTCFs in Québec active (providing health and personal care services) at any time between May 2022 and September 2024, totaling 471 LTCFs (93.2% were active during the entire study period) and 45,653 beds. Provincial Nosocomial Infection Surveillance Information System (*Système d’information sur la surveillance provinciale des infections nosocomiales* or SI-SPIN)^23^ data on COVID-19 active outbreaks were used, aggregated by LTCF and surveillance week (designated as epiweek).

The study period was divided into three distinct surveillance periods defined as follows: May 29th, 2022 - September 3rd, 2022 (wave 7), September 4th, 2022 - August 26th, 2023 (2022-23 season), and August 27th, 2023 - September 21st, 2024 (2023-24 season). Although SARS-CoV-2 seasonality is not established, we followed respiratory viruses’ seasons starting from the 2022-23 season. Data extraction was performed on September 23rd, 2024. Outbreaks that were still active on that date were censored. Data entry in this surveillance system is very rapid (within a week). For COVID-19 weekly cases in the general population (all age groups), we used data from Québec’s COVID-19 surveillance system^24^.

Omicron BA.4, BA.5* and BQ.1 were the predominant variants during wave 7, while the other two periods had different co-circulating variants (BQ.1*, XBB.1.5*, XBB.1.9* during the 2022-23 season; JN.1*, KP.2*, KP.3* during the 2023-24 season; see Fig. S1).

### Variables

#### Definition of COVID-19 case in a LTCF

A COVID-19 case (including residents and HCWs) was identified through either laboratory testing (polymerase chain reaction or PCR), rapid antigen detection test (RAT), or when an epidemiological link was established with another lab-confirmed or probable (RAT or epidemiological link) case^25,26^. An epidemiological link is established when a criterion of time, place or person is compatible with transmission from a confirmed case to a susceptible individual^17^. Specifically, an epidemiological link is established if users (residents or HCW) stayed in the same environment (e.g., room) less than two meters away and without barrier measures in place at any time during the contagious period of the COVID-19 case for 10 min (the criteria of 10 min does not apply between residents). The definition of an LTCF-acquired SARS-CoV-2 infection was modified during the study period. Before July 19th, 2023, a SARS-CoV-2 infection was considered acquired in the LTCF when it occurred 7 days or more after admission. After that date, the delay was shortened to 72 h to account for the shortened incubation period^26^.

Starting on July 19th, 2023, SARS-CoV-2 screening tests were no longer available for asymptomatic LTCF residents at admission. Asymptomatic LTCF residents were tested if they shared a room with a COVID-19 patient^27^. Asymptomatic HCWs were tested if they had a close contact with a confirmed case during an outbreak. Definitions for COVID-19-like symptoms are provided in Table S1^28^. A timeline of SARS-CoV-2 testing indications in LTCFs is provided in Table S2^29–35^.

#### SARS-CoV-2 outbreak definition

An outbreak was defined as two cases of COVID-19 acquired in an LTCF unit (including both residents and HCWs) occurring within a 10-day period (14 days prior to July 19th, 2023) and linked epidemiologically^25^. An outbreak was considered over if no new cases linked to the outbreak had been reported in the last 10 days. If two or more outbreaks in different units overlapped in time within the same LTCF, they were treated as a single outbreak for this study, since bed-days were available by LTCF rather than by care unit.

#### Definition of COVID-19 case in the general population

All general population cases were laboratory-confirmed by PCR. The population eligible for SARS-CoV-2 detection tests was inconsistent over the study period, targeting both symptomatic and asymptomatic individuals (unknown proportions). Of note, reported cases among the general population comprise SARS-CoV-2 outbreak cases, including LTCFs, hospitals and private seniors’ residences^36^.

#### Definition of COVID-19 death cases

Any death among LCTF COVID-19 cases that occurred during an outbreak was considered as a COVID-19 death.

### LTCF characteristics

Québec health regions were grouped as follows: Montréal, the Greater Montréal Area (*Montérégie*,* Lanaudière*,* Laurentides*,* Laval*), other urban regions (*Capitale-Nationale*,* Mauricie-Centre-du-Québec*,* Estrie*,* Outaouais*,* Chaudières-Appalaches*,* Saguenay-Lac-St-Jean*) and less populated regions (*Bas-St-Laurent*,* Abitibi-Témiscamingue*,* Côte-Nord*,* Nord-du-Québec*,* Gaspésie-Îles-de-la-Madeleine*,* Terres-Cries-de-la-Baie-James and Nunavik*). This grouping is based on population density, proximity to Montréal and level of urbanicity. LTCF bed capacity was categorized by number of beds as follows: 10–39; 40–64; 65–99; 100–149; ≥150^9^.

### Statistical analyses

For descriptive analysis, we reported the proportion of LTCF with at least one outbreak, the median outbreak duration, the median outbreak size and the median weekly associated cases in all LTCFs stratified by surveillance period, regions and LTCF bed-capacity. The cross-sectional association between the number and size (including both residents and HCWs cases) of outbreaks with LTCF characteristics was assessed using the Wilcoxon rank sum test.

The optimal lag between the weekly number and size of outbreaks in LTCFs (outcomes) and weekly COVID-19 cases in the general population (exposure variable) was identified through cross-correlation analysis. Linear regression models were then used to estimate association between the outcomes and the exposure variable for the three periods (wave 7, 2022-23 season and 2023-24 season)^37^. To compare the level of association between COVID-19 outbreaks and associated cases with COVID-19 cases in the general population across the three periods, models with an interaction term between total number of COVID-19 in Québec and periods were used.

Logistic regression models were applied to assess the odds ratio of an active outbreak in LTCFs at any given week over periods (reference: wave 7), region (reference: Montréal) and LTCF bed capacity (reference: 10–39 beds); generalized estimating equations (GEE) were used to account for repeated observations per LTCF. GEE Poisson regression models were applied to measure the association between COVID-19 case incidence rates in LTCFs with aforementioned characteristics, using the log of the number of bed-days as the offset. A sensitivity analysis included only cases among LTCF residents, i.e. excluding cases among HCWs. Both logistic and Poisson regression were carried out using both censored and uncensored outbreaks.

GEE logit-binomial regression models were used to measure the association between CFR in LTCFs (only for uncensored outbreaks and including only residents) and the aforementioned characteristics.

Multivariate analyses always included all adjustment variables.

Multiple covariance matrix structures (exchangeable, independent and autoregressive) were tested for the GEE models. Their performance was assessed using the Quasi-likelihood under the Independence model Criterion (QIC), with the lowest QIC value indicating the optimal correlation structure^38^. The independent structure was deemed optimal and applied to all regression models.

All statistical analyses were carried out using R Studio software (2024.12.0 Build 467). Type I error risk was 0.05. Results were presented as point estimates with 0.95 confidence intervals.

## Results

A total of 2,501 outbreaks were recorded, corresponding to 39,089 COVID-19 associated outbreak cases between May 29, 2022 (epiweek 22) and September 21st, 2024 (epiweek 38). Of these, 83.3% (*n* = 32,581) were outbreaks-associated cases among residents and 16.7% (*n* = 6,508) among HCWs. About 94.6% of outbreaks-associated cases (*n* = 36,997) were confirmed by PCR, 4.5% (*n* = 1,754) by rapid antigenic test and 0.9% (*n* = 338) by epidemiological link.

As of September 21st, 2024, 95 active outbreaks-associated with 1063 COVID-19 cases were identified and censored. Median number of cases among censored outbreaks was 8 [interquartile range: 5–14].

**Fig. 1:**
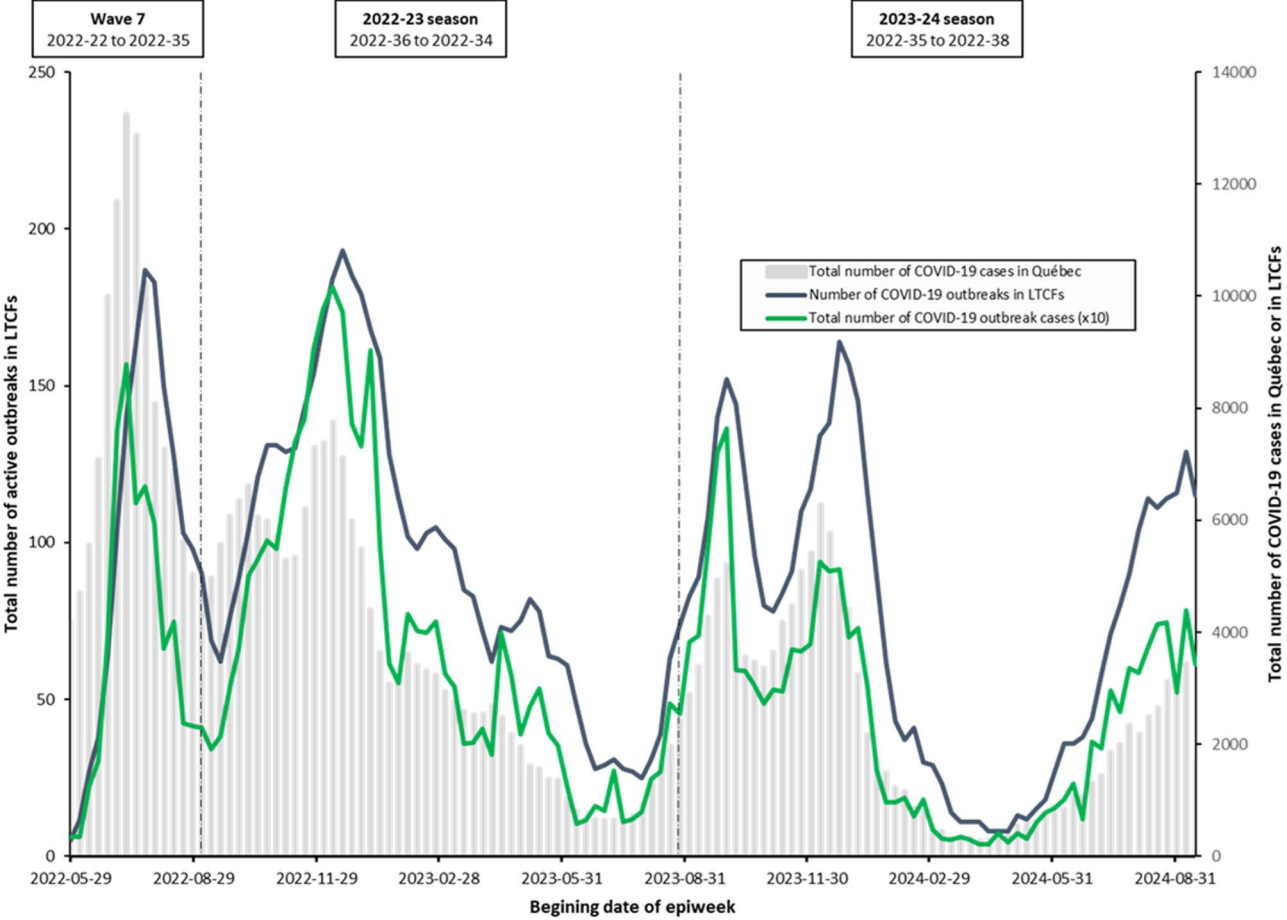
Weekly number of COVID-19 active outbreaks in long-term care facilities and total weekly number of outbreaks associated COVID-19 cases and Québec, Canada (epiweek 22-2022 to 38-2024). Total number of COVID-19 cases among the general population in Québec includes COVID-19 outbreak cases in LTCFs, hospitals, private seniors’ residence, and other settings. The number of COVID-19 cases in LTCFs was rescaled (multiplied by 10) to match the same range of values as the total number of COVID-19 cases in the province, allowing them to be displayed on the same y-axis.

Figure 1 shows the trend of weekly active outbreaks along with COVID-19 cases in the general population.

### COVID-19 outbreak characteristics

Only 54% of LTCFs had at least one outbreak during wave 7, which was expected as this surveillance period was shorter than the other two periods (Table 1). The proportion of LTCFs with at least one outbreak was around 90% for the other two surveillance periods.

**Table 1:** COVID-19 outbreaks in long-term care facilities in Québec, Canada (epiweek 22-2022 to 38-2024).

LTCF characteristic	LTCF with ≥ 1 outbreak, *N* (%)	Outbreak duration^a^ (days), median [IQR]	Size of outbreaks^a, b^, median [IQR]	Weekly cases in all LTCFs, median [IQR]
Total	446 (94.5)	20 [15–27]	10 [5–20]	298 [121–418]
Surveillance period^c^				
Wave 7	238 (53.8)	22 [17–28]	11 [6–22]	382 [186–621]
2022-23 season	400 (89.5)	24 [19–31]	13 [6–26]	303 [194–540]
2023-24 season	415 (88.5)	17 [12–23]	8 [4–17]	282 [77–375]
Region				
Montréal	92 (96.8)	21 [15–28]	11 [5–21]	65 [22–118]
Greater Montréal area	51 (91.1)	20 [15–26]	10 [5–21]	22 [8–43]
Other urban regions	179 (96.7)	20 [15–26]	10 [5–20]	102 [40–163]
Less populated regions	124 (93.9)	21 [15–28]	11 [5–22]	84 [32–133]
LTCF bed capacity^d^				
10–39	81 (88.0)	19 [15–23]	8 [5–16]	14 [5–29]
40–64	86 (93.4)	20 [15–26]	9 [5–18]	33 [10–55]
65–99	113 (94.9)	20 [15–27]	10 [5–20]	57 [20–104]
100–149	82 (100)	20 [15–28]	10 [5–23]	64 [25–101]
≥ 150	83 (98.8)	21 [15–31]	11 [5–25]	101 [54–148]

^a^Only outbreaks that were over as of September 21st, 2024, were considered. ^b^The outbreak size is based on the number of reported cases, including LTCF residents and health workers. ^c^Period corresponding to the end of the outbreak for the proportion of LTCFs which experienced at least one outbreak, outbreak size and duration. For the weekly COVID-19 cases in all LTCFs, the period was defined based on the surveillance week. ^d^Bed capacity was unavailable for one LTCF on September 21st, 2024.

Median outbreak duration was 20 days over the entire study period. It increased during the 2022-23 season (24 days vs. 22 days during wave 7, *p* = 0.0033) but then dropped to its lowest level during the 2023-24 season (17 days, *p* < 0.0001) (Table 1 and Fig. S2). The outbreak duration increased with bed capacity, with 21 days for those with ≥ 150 beds compared to 19 days for those with 10–49 beds (*p* < 0.0001).

The median number of reported COVID-19 cases (residents and HCWs) per outbreak was 10 [IQR: 5–21] over the entire study period. Compared to wave 7, outbreak size did not change in the 2022-23 season (*p* = 0.11) but and, again, declined in the 2023-24 season (*p* < 0.0001). Outbreak size increased with LTCF bed capacity (8 cases during wave 7 vs. 11 cases during 2023-24 season, *p* = 0.0002).

The median number of weekly new outbreaks associated COVID-19 cases in all LTCFs over the entire study period was 298 [IQR: 121–418]. Despite a downward trend in the median number of weekly outbreaks-associated cases, there was no significant difference between wave 7 and the 2023-24 season (*p* = 0.14). Median weekly outbreak-associated cases were significantly lower in the 2023-24 season than in the 2022-23 season (*p* = 0.047), while significant differences were found between regions (Fig. S2).

### Correlation between COVID-19 outbreaks in LTCFs and associated cases and COVID-19 cases in the general population

There was a significant correlation (*R* ≥ 0.89; *p-*values < 0.0001) between the weekly number of COVID-19 active outbreaks in LTCFs and COVID-19 cases in the general population. A significant correlation (*R* ≥ 0.85; *p-*values < 0.0001) was also observed between the number of outbreaks-associated COVID-19 cases in LTCFs and COVID-19 cases in the general population. For the weekly number of outbreaks in LTCFs, the slopes observed during wave 7 and 2022-23 season were comparable (*p* = 0.69); however, the slope of wave 7 was lower than that of 2023-24 season (*p* = 0.023). For weekly outbreaks-associated COVID-19 cases in LTCFs, all slopes were comparable (*p* = 0.42 for wave 7 vs. 2022-23 season; *p* = 0.34 for wave 7 vs. 2023-24 season). An increase of 5,000 weekly cases in the general population was associated with 53, 119, and 140 additional outbreaks (Fig. S3), and 168, 532, and 508 additional cases in LTCFs (Fig. S4) during wave 7, the 2022-23 season and the 2023-24 season, respectively.

### Association between LTCF characteristics, period of study and active COVID-19 outbreak status

Odds of an active outbreak during the 2022-23 season were comparable to those in wave 7 (adjusted OR = 0.91 [95% CI: 0.81–1.03]) but were smaller during the 2023-24 season compared to wave 7 (adjusted OR = 0.65 [95% CI: 0.58–0.73]) (Table 2).

**Table 2:** Logistic regression for the association between the COVID-19 active outbreak status in long-term care facilities and surveillance period, region, and bed capacity (epiweek 22-2022 to 38-2024), Québec, Canada.

	Cumulative number of LTCF-week		Bivariate analysis		Multivariate analysis	
	with COVID-19 outbreaks (*n*)	without COVID-19 outbreaks (*n*)	Odds ratio^a^	95% CI^b^	Odds ratio	95% CI^b^
Surveillance period^c^						
Wave 7	1,388	4,783	1		1	
2022-23 season	4,755	1,7838	0.92	0.82–1.03	0.91	0.81–1.03
2023-24 season	4,170	21,572	0.67	0.60–0.75	0.65	0.58–0.73
Region						
Montréal	2,578	8,829	1		1	
Greater Montréal Area	883	5,783	0.52	0.40–0.69	1.11	0.91–1.35
Other urban regions	3,800	17,826	0.73	0.60–0.88	1.21	1.07–1.38
Less populated regions	3,052	11,755	0.89	0.73–1.09	1.15	0.99–1.32
LTCF bed capacity						
10–39	814	10,327	1		1	
40–64	1,409	9,092	1.97	1.65–2.34	1.97	1.65–2.35
65–99	2,379	11,029	2.74	2.32–3.22	2.78	2.35–3.29
100–149	2,267	7,249	3.97	3.36–4.69	4.13	3.46–4.93
≥ 150	3,444	6,496	6.73	5.67–7.98	7.17	6.00–8.58

^a^The bivariate analysis includes each of the adjustment variable separately. ^b^CI: confidence interval. ^c^Period corresponding to the end of the outbreak for the proportion of LTCFs which experienced at least one outbreak, outbreak size and duration. For the weekly COVID-19 cases in all LTCFs, the period was defined based on the surveillance week.

LTCFs located in ‘*Other urban and less populated regions*’ were more likely to report an outbreak during the study period compared to those in the Montréal region (adjusted OR = 1.21 [95% CI: 1.07–1.38]). Large (≥ 150 beds) LTCFs were 7.23 times (95% CI) more likely to report an active outbreak compared to smaller (10–39 beds) LTCFs.

### Association between outbreak-associated COVID-19 cases incidence rate ratio in LTCFs and characteristics

During the 2023-24 season, outbreak-associated COVID-19 cases adjusted incidence rate ratio (IRR) decreased by 39% [95% CI: 31–46] compared to wave 7, while the 2022-23 season showed a comparable incidence rate (adjusted IRR = 0.96 [95% CI: 0.84–1.09]) (Table 3). Adjusted IRR were significantly higher in all regions (from + 21% to + 39%; *p* values < 0.0001) compared to Montréal. There was no association between COVID-19 adjusted IRR and LTCF bed capacity (multivariate risk reduction= -9% to + 5%; *p* values > 0.05). A sensitivity analysis including only outbreak-associated cases in LCTF residents and excluding cases in HCWs yielded similar results (Table S3).

**Table 3:** Poisson regression for the association between the incidence rate ratio of outbreak-associated COVID-19 cases in long-term care facilities and surveillance period, region, and bed capacity (epiweek 22-2022 to 38-2024).

	Number of events	Number of weeks	Number of bed-days	Bivariate analysis^a^		Multivariate analysis	
				IRR^b^	95% CI^c^	IRR^b^	95% CI^c^
Surveillance period^d^							
Wave 7	5,520	14	4,236.022	1		1	
2022-23 season	19,440	51	15,529.969	0.96	0.84–1.09	0.96	0.84–1.09
2023-24 season	13,988	56	17,600.562	0.61	0.53–0.69	0.61	0.54–0.69
Region							
Montréal	9,922	121	1,633.016	1		1	
Greater Montréal Area	3,444	121	389,846	1.45	1.26–1.68	1.39	1.20–1.61
Other urban regions	14,074	121	1,778.967	1.30	1.19–1.42	1.26	1.15–1.39
Less populated regions	11,508	121	1,536.250	1.23	1.12–1.35	1.21	1.10–1.33
LTCF Bed capacity							
10–39	2,408	121	2,059.533	1		1	
40–64	4,707	121	3,889.634	1.04	0.88–1.21	1.05	0.91–1.22
65–99	8,497	121	7,751.604	0.94	0.81–1.08	0.98	0.85–1.12
100–149	8,598	121	8,204.343	0.90	0.77–1.04	0.95	0.82–1.11
≥ 150	14,738	121	15,461.439	0.82	0.71–0.94	0.91	0.79–1.04

^a^The bivariate analysis includes each of the adjustment variable separately with the offset variable. ^b^Incidence rate ratio. ^c^CI: 0.95 confidence interval. ^d^Period corresponding to the end of the outbreak for the proportion of LTCFs which experienced at least one outbreak, outbreak size and duration. For the weekly COVID-19 cases in all LTCFs, the period was defined based on the surveillance week.

### Association between case fatality ratio in LTCFs and characteristics

Compared to wave 7, the CFR was 18% [95% CI: 1–33] and 24% [95% CI: 8–38] lower during the 2022-23 and 2023-24 seasons, respectively (Table 4). Higher CFR were found in all regions (adjusted ratio of CFRs = 1.88 to 2.08; *p* values < 0.0001) compared to Montréal, but there was no statistically significant difference in CFR observed across LTCF bed capacity categories.

**Table 4:** Log-binomial regression for the association between case-fatality ratio (CFR) and surveillance period, region, and long-term care facilities bed capacity (epiweek 22-2022 to 38-2024).

	COVID-19 deaths (*n*)	Case-fatality ratio	Bivariate analysis^a^		Multivariate analysis	
			Ratio of CFR	95% CI^b^	Ratio of CFR	95% CI^b^
Surveillance period^c^						
Wave 7	207	4.4	1		1	
2022-23 season	590	3.6	0.82	0.67–0.98	0.82	0.67–0.99
2023-24 season	352	3.4	0.76	0.62–0.93	0.76	0.62–0.92
Region						
Montréal	186	2.2	1		1	
Greater Montréal Area	108	4.5	2.04	1.48–2.82	2.08	1.46–2.93
Other urban regions	469	4.2	1.90	1.57–2.30	1.91	1.56–2.35
Less populated regions	386	4.1	1.84	1.49–2.27	1.88	1.51–2.34
Bed capacity						
10–39	73	4.2	1		1	
40–64	146	4.1	0.97	0.70–1.35	0.96	0.69–1.33
65–99	254	3.8	0.89	0.67–1.19	0.92	0.68–1.25
100–149	245	3.5	0.84	0.63–1.12	0.93	0.68–1.25
≥ 150	431	3.4	0.81	0.62–1.08	0.99	0.74–1.32

^a^The bivariate analysis includes each of the adjustment variable separately. ^b^CI: 0.95 confidence interval. ^c^Period of beginning of the COVID-19 outbreak.

## Discussion

This is a comprehensive study of all COVID-19 outbreaks, outbreak-associated COVID-19 cases, and COVID-19 deaths that occurred in LTCFs from May 2022 to September 2024 in Québec, Canada. Over those two years, 2,501 outbreaks and 39,089 COVID-19 cases were recorded, representing 9.2% of total cases (*n* = 423,899) reported in Québec among the general population for the same period. Nearly all LTCFs faced at least one outbreak during the study period. We found that the odds of experiencing an active COVID-19 outbreak on any given week, associated COVID-19 cases incidence and CFRs (COVID-19 deaths) all declined over the study period.

The odds of outbreaks and incidence rate of outbreaks-associated COVID-19 cases declined significantly during the 2023-24 season compared to wave 7. Several factors could have contributed to the phenomenon, such as virological properties of the circulating Omicron variants (e.g. S protein mutations at the N-terminal domain (NTD) and receptor-binding domain)^39,40^, updated vaccination^41^, high vaccine coverage against SARS-CoV-2^42^, and the development of population-level immunity from previous infections^43^. Despite increased evasion of immune response and higher transmission fitness of successive Omicron variants^44^, transmission might have been hampered by a relative decrease of viral shedding period, which was observed for BA.1 and BA.2 compared to Delta^45^. However, the decline of viral shedding period may have been inconsistent during the study period^46^.

Contextual factors might have also contributed to the reported declining trends of COVID-19 in LTCFs. For example, COVID-19 screening tests were unavailable for asymptomatic LTCF residents starting from July 2023, except for those considered close contacts during an outbreak (more details are available in Table S2). This change might have resulted in an under-reporting of COVID-19 outbreaks and associated cases in LTCFs because a significant proportion of elderly individuals could have acquired an asymptomatic or paucisymptomatic SARS-CoV-2 infection despite an ageing immune system and/or the presence of chronic health conditions; this still reflects an improvement of the situation^47^. A study conducted in Belgium during the beginning of the pandemic (April 8th to May 18th, 2020) showed that 75% of LTCF residents with a positive PCR test were asymptomatic^47^. Although the cross-sectional design of the survey may have led to an underestimation of the prevalence of residents who may develop symptoms later, a significant proportion of asymptomatic individuals may still be present. Considering that the Omicron variants cause less severe disease than pre-Omicron variants, it is expected that the number of asymptomatic cases would have risen since the Omicron predominance period, which would increase the underreporting^48^. Finally, it is unlikely that the declining trend in outbreaks-associated COVID-19 cases can be attributed to the exclusion of most asymptomatic HCWs at the mid-study period. A sensitivity analysis showed that the results stand when including only LTCF residents and excluding HCWs (Table S3).

We reported a decline in CFR during the study period. This finding is consistent with population-level surveillance reports in Québec^49^, other Canadian provinces^50^, and other countries^51,52^. For instance, in Canada and the United States, deaths where COVID-19 is listed as an underlying or contributing cause declined from 2022 to 2023 among those aged 65 years and older by nearly 58%^53^ and 65%^54^, respectively. Globally, according to the World Health Organization, deaths per 1000 hospitalizations declined from 253 in June 2021 to 41 by November 2024^51^. Studies carried out since the emergence of the BA.5 variant and onward have shown a relatively stable symptoms presentation and comparable or lower severity of illness over time^55–58^. However, it is worth mentioning that COVID-19 incidence rate is most likely subject to surveillance bias^59^. This bias could be observed because users were no longer tested unless they were a close contact of a COVID-19 case starting from the mid-study period. Then, the surveillance bias might have increased the underreporting of cases, decreasing the incidence of COVID-19 outbreak and associated cases; however, our case definition of symptomatic patient is broad (Table S1), allowing the detection of paucisymptomatic residents. For example, a resident with nasal congestion and headache could be systematically being tested for SARS-CoV-2 infection. Also, our case definition includes atypical symptoms such as nausea, vomiting, and abdominal pain which are frequent (29%) among older adults^60^. This would also apply to COVID-19 deaths, but to a lesser extent and despite this, a decreasing CFR was observed^59^.

Larger LTCF bed capacity was associated with increased odds of an active outbreak. LTCFs with a bed capacity of 150 or more were about seven times more likely to report an active outbreak at a given time than those with 10–39 beds over all study periods. One of the hypotheses would be that larger LTCFs are more prone to imported COVID-19 cases from the general population than smaller ones, as they tend to have more visitors (restrictions had been progressively relaxed over time between 2022 and 2024) and caregivers^61,62^. For example, a pre-Omicron study by Orlando et al. reported that LTCFs with ≥ 15 beds had 5.3 times [95% CI: 1.58–22.8] higher odds of reporting an outbreak compared to those with less than 15 over the study period^61^. Similarly, Lambardo et al. found that LTCFs with > 60 beds were 1.57 times (95% CI: 1.17–2.09) more at risk of experiencing an outbreak compared to those with ≤ 60 beds between March and April 2020^62^. However, even though larger LTCFs were more likely to report an outbreak during the study period, COVID-19 outbreak cases incidence were comparable. Even if larger LTCFs have probably more imported infections (visitors and HCW), SARS-CoV-2 transmission within facilities remained comparable across LTCF bed capacity. The stringent application of the preventive and control measures in the different LTCFs might have contributed to limiting the spread of the virus once it reached a facility.

Interestingly, outbreaks-associated COVID-19 cases incidence rate and CFR were not associated with LTCF bed capacity, while during the first pandemic wave (February 23rd to July 11th, 2020), LCTF bed capacity was strongly correlated with COVID-19 cases incidence rate^9^. During the first two months of the second wave (August 23rd to November 21st, 2020), incidence rates were relatively comparable across LTCF bed capacity^9^. Infection prevention and control measures (hand washing, use of personal protective equipment, environment disinfection, proving single isolation room, if possible, with all amenities, and standardised procedure for the management of outbreaks) probably mitigated the association between LTCF bed capacity and virus transmission; in other word irrespective of the LTCF bed capacity, the transmission of the virus was similarly controlled.

### Strengths

Our study includes all LTCFs located in Québec, thereby maximizing representativeness. Most outbreaks-associated COVID-19 cases (95%) were identified using PCR tests rather than rapid antigen tests, ensuring high validity. While the ecological study design remains limited for causal inference at the individual level, it remains appropriate to follow trends over time.

### Limitations

First, the eligibility for SARS-CoV-2 screening was inconsistent during the study period. Indeed, during the mid-period of the study, asymptomatic residents and HCW (with few exceptions as mentioned previously) (Table S2) were no longer tested. Second, only COVID-19 cases identified during an outbreak were reported here, thus sporadic cases were not considered. This would underestimate the total incidence of COVID-19 cases in LTCFs, but this bias is presumed to be consistent throughout the study period. Third, censored outbreaks were included while analysing the association between outbreak-associated cases incidence and LTCF characteristics. The analysis was rerun in February 2025 with complete data (*detailed data not shown*), and associations remained consistent. Therefore, the missed cases did not bias our analysis. Forth, starting from July 2023, definitions of an LTCF-acquired SARS-CoV-2 infection and an outbreak were adapted: (i) an infection was considered as LTCF-acquired if the resident tested positive ≥ 3 days after admission (instead of ≥ 7 days prior to July 2023). New admissions in LTCFs are less frequent than in acute care settings, so imported cases from such events are expected to be rare. Consequently, it is unlikely that changes in the LTCF-acquired infection definition for new arrivals would have a significant impact on reported trends; (ii) The time interval without any new case required to close an outbreak was shortened from 14 to 10 days. It is unlikely that this shorter time interval (10 days) will lead to misleadingly declaring an outbreak over, as the viable virus shedding time (5 days from the symptoms onset^45^ remains far shorter. However, probably this adjustment of the time interval to 10 days could underestimate the CFR, which is defined based on the LTCF COVID-19 deaths that occurred while the outbreak is still active.

Information on the crowding index was not available. However, the occupancy proportion of LTCFs in Québec (either public or private) is, on average, very high (97%)^22^, suggesting low crowding variability across LCTFs. Also, other predictors of COVID-19 cases were not available, e.g. the number of HCW per resident^62^ and other LTCF characteristics (e.g. design and use of multi-occupant rooms)^63^. Finally, also because the currently admitted residents are older, frail adults having lower functional levels. Then, even if information on the health status of admitted residents in LTCFs is limited, we are not expecting a significant variability across different settings.

## Conclusion

In summary, 95% of the LTCFs have experienced at least one outbreak during the study period. We reported a progressive decline in active outbreak odds, associated COVID-19 cases and deaths in LTCFs from May 2022 to September 2024. The death burden in LCTFs declined significantly compared to the first year of the pandemic, before vaccines were first administered in this population. As expected, larger LTCFs were more likely to have an active outbreak at any given time during the study period, while incidence rate of outbreaks-associated COVID-19 cases within facilities were comparable, irrespective of LTCFs’ bed capacity. This suggests that probably the preventive and control measures were implemented in a comparable manner, which reduced variability of SARS-CoV-2 transmission within LTCFs irrespective of their bed capacity.

## Supplementary Information

Below is the link to the electronic supplementary material.

Supplementary Material 1

## Data Availability

These datasets generated and/or analysed during the current study are not publicly available because they are a property of the Ministère de la Santé et des Services sociaux du Québec (MSSS); but are available from the MSSS upon reasonable request and with permission. The access can be requested from the Direction Générale de la gouvernance et des affaires institutionnelles through the following email address [responsable.acces@msss.gouv.qc.ca]. More details are available through the following link: https://www.quebec.ca/en/gouvernement/ministeres-organismes/sante-services-sociaux/cadre-legal-transparence/access-information-protection-personal-information.
