# Supplementary material for: SARS-CoV-2 outbreaks in long-term care facilities during the Omicron era in Québec, Canada
Source: Sci Rep. 2025 Dec 23;16:3122. doi: 10.1038/s41598-025-32967-y (PMC12830647; doi:10.1038/s41598-025-32967-y)
Supplement: Supplementary file 1 — Supplementary Material 1 [file 41598_2025_32967_MOESM1_ESM.docx]

**Table S1.** Definition of symptomatic residents^28^

Symptomatic residents are defined as follow:

- Any of the following symptoms:
  - Fever
  - Cough (new or worsened)
  - Sore throat
  - Difficulty breathing or shortness of breath (dyspnea)
  - Anosmia or agueusia or dysgeusia

OR two of the following symptoms:

- Significant loss of appetite
- Intense fatigue
- Generalized muscle pain (not related to physical exertion)
- Unusual headache
- Diarrhea
- Nausea or vomiting
- Abdominal pain
- Rhinorrhea or nasal congestion of unknown cause

**Table S2.** Timeline of COVID-19 testing indications in long term care facilities (including health care workers and residents) ^29–36^

| **Date of update** | **Directive from the MSSS or the recommendations of the INSPQ** | **Testing strategy in long-term care facilities** |
| --- | --- | --- |
| May 25^th^, 2022 | [DGSP-018.REV10](https://publications.msss.gouv.qc.ca/msss/document-003396/?&txt=DGSP-018&msss_valpub&index=directives-covid-19&date=DESC) ^31^ | - Asymptomatic HCWs with close contact during an outbreak: Screening with PCR on day 5 after last exposure, unless previous episode of COVID-19 ≤ 60 days. - Symptomatic HCWs:   - Not tested if had a previous COVID-19 infection within the last ≤60 days.   - Tested if not having prior infection or had COVID-19 infection >60 days: PCR (repeated within 24 hours if had persistent symptoms) |
| July 25^th^, 2022 | [DGSP-018.REV11](https://publications.msss.gouv.qc.ca/msss/document-003446/?&txt=DGSP-018&msss_valpub&index=directives-covid-19&date=DESC) ^30^ | *Same strategy as the precedent directive*. However, in case of compromised care and basic services, teams are allowed to adapt the frequency and timing of screening of asymptomatic HCWs who have been in close contact. |
| December 21^th^, 2022 | [DGSP-001.REV9](https://publications.msss.gouv.qc.ca/msss/document-003517/) ^32^ | HCWs in contact with patients/users with COVID-19 like symptoms. |
| [July 19^th^, 2023](https://www.cisss-lanaudiere.gouv.qc.ca/fileadmin/internet/cisss_lanaudiere/Professionnels/Biologie_medicale__laboratoires_/Biologie_medicale_Sud/Serologie_et_virologie/Tableau_des_indications_d_acces_aux_TAAN__tests_PCR__2023-07-19.pdf) | MSSS ^29^ | - HCWs are tested:   - If had contact with residents having COVID-19 like symptoms.   - If attending to confirm a positive result to a RAT or any other self-administered test. - Asymptomatic HCWs Asymptomatic are tested if they had a close contact during an outbreak. |
| June 4^th^, 2024 | MSSS | Same strategy as of July 19^th^, 2023 |
| May 13^th^, 2022 | [DGCRMAI-004.REV3](https://publications.msss.gouv.qc.ca/msss/document-003369/) ^33^ | - Any resident is tested at the admission (PCR or RAT) - Asymptomatic: tested if had any contact with COVID-19 case at day 0 (also day 4 and day 5 if it was a close contact) - Symptomatic: Tested at day 0 and within 24 to 36h |
| July 25^th^, 2022 | [DGCRMAI-004.REV4](https://publications.msss.gouv.qc.ca/msss/fichiers/directives-covid/archives/dgcrmai-004-rev4.pdf) ^34^ | - Any resident is tested at the admission (PCR or RAT) - Asymptomatic residents with close contact to a positive COVID-19 case: - Tested at day 0, day 4-5 and had no previous infection or have been infected with COVID-19 more that 3 months ago. - Tested at day 0 and day 9-10 if: i) COVID-19 infection >6 months ago AND unvaccinated; ii) COVID-19 infection >12 months ago AND incomplete primary vaccination; iii) No confirmed COVID-19 infection AND unvaccinated or incomplete primary vaccination; iv) Immunosuppressed person, vaccinated or not, previous COVID-19 confirmed or not; v) Refusal of COVID-19 screening. - Not tested if an infection confirmed by PCR, rapid antigen test, or epidemiological link during the last 3 months - Symptomatic: Tested at day 0 and within 24 to 36h |
| December 12^th^, 2022 | [DGCRMAI-004.REV5](https://publications.msss.gouv.qc.ca/msss/document-003516/) ^28^ | - Asymptomatic residents with close contact to a positive COVID-19 case: - Tested (PCR or RAT) at day 0, day 4-5 and had no previous infection or have been infected with COVID-19 more that 3 months ago. - Tested (PCR or RAT) at day 0 and day 9-10 if: i) COVID-19 infection >6 months ago AND unvaccinated; ii) COVID-19 infection >12 months ago AND uncompleted primary vaccination schedule (2 doses of vaccine licensed by Health Canada, or 3 doses for immunocompromised or dialyzed individuals) ; iii) No confirmed COVID-19 infection AND unvaccinated or incomplete primary vaccination; iv) Immunosuppressed person, vaccinated or not, previous COVID-19 confirmed or not; v) Refusal of COVID-19 screening. - Not tested if had an infection confirmed by PCR, rapid antigen test, or epidemiological link during the last 3 months - Symptomatic: Tested at day 0 and within 24 to 36h |
| April 6^th^, 2023 | [DGCRMAI-004.REV6](https://publications.msss.gouv.qc.ca/msss/fichiers/directives-covid/archives/dgcrmai-004-rev6.pdf) ^35^ | - Asymptomatic residents with a contact with COVID-19 case but defined as no close: Tested at day 0 if the source of the exposure is unknown - Asymptomatic residents with close contact to a positive COVID-19 case: - Tested at day 0, day 4-5, day 7 (only for those with uncompleted basic vaccination (without prior infection: 2 primary vaccination doses and 1 booster dose; with prior infection: 2 primary vaccination doses) or refused to be tested) and had no previous infection or have been infected with COVID-19 more that 3 months ago. - Not tested if had a confirmed by PCR, rapid antigen test, or epidemiological link during the last 3 months except for those immunocompromised. - Symptomatic: Tested at day 0 and within 24 to 36h |
| [July 19^th^, 2023](https://www.cisss-lanaudiere.gouv.qc.ca/fileadmin/internet/cisss_lanaudiere/Professionnels/Biologie_medicale__laboratoires_/Biologie_medicale_Sud/Serologie_et_virologie/Tableau_des_indications_d_acces_aux_TAAN__tests_PCR__2023-07-19.pdf)^29^ | - | - Symptomatic LTCF residents only. - Asymptomatic residents were no longer tested at the admission. Asymptomatic LTCF residents were tested if they had a close contact with a COVID-19 patient (sharing the same room) |
| June 4^th^, 2024 |  | Same strategy as of July 19^th^, 2023. |

HCWs: Health care workers; LTCF: long term care facilities; MSSS: Ministère de la Santé et des Services sociaux; PCR: polymerase chain reaction; RAT: rapid antigenic test. NB: blue color refers to directives regarding HCWs.

**Table S3.** Multivariate Poisson regression for the association between the incidence of COVID-19 cases among LTCF residents and surveillance period, region, and bed capacity (epiweek 2022-22 to 2024-38).

|  | **Incidence rate ratio** | **95 % CI**^a^ |
| --- | --- | --- |
| **Surveillance period** |  | |
| *Wave 7* | Ref |  |
| *Season 2022-23* | 0.98 | 0.86 – 1.12 |
| *Season 2023-24* | 0.62 | 0.54 – 0.70 |
| **Region** |  | |
| *Montréal* | Ref |  |
| *Ring of Montréal* | 1.24 | 1.08 – 1.43 |
| *Other urban regions* | 1.21 | 1.10 – 1.33 |
| *Less populated regions* | 1.20 | 1.09 – 1.32 |
| **Bed capacity** |  | |
| *10 – 39* | Ref |  |
| *40 – 64* | 1.09 | 0.94 – 1.26 |
| *65 – 99* | 1.05 | 0.92 – 1.20 |
| *100 – 149* | 1.04 | 0.90 – 1.19 |
| *≥150* | 1.02 | 0.89 – 1.17 |

^a-^CI: 0.95 confidence interval.

**Fig. S1.** Omicron sublineages prevalence for available sequence data in the province of Québec (Canada) with a specimen date from week beginning May 29^th^, 2022, to week beginning September 15^th^, 2024, as of January 31^st^, 2025.

**
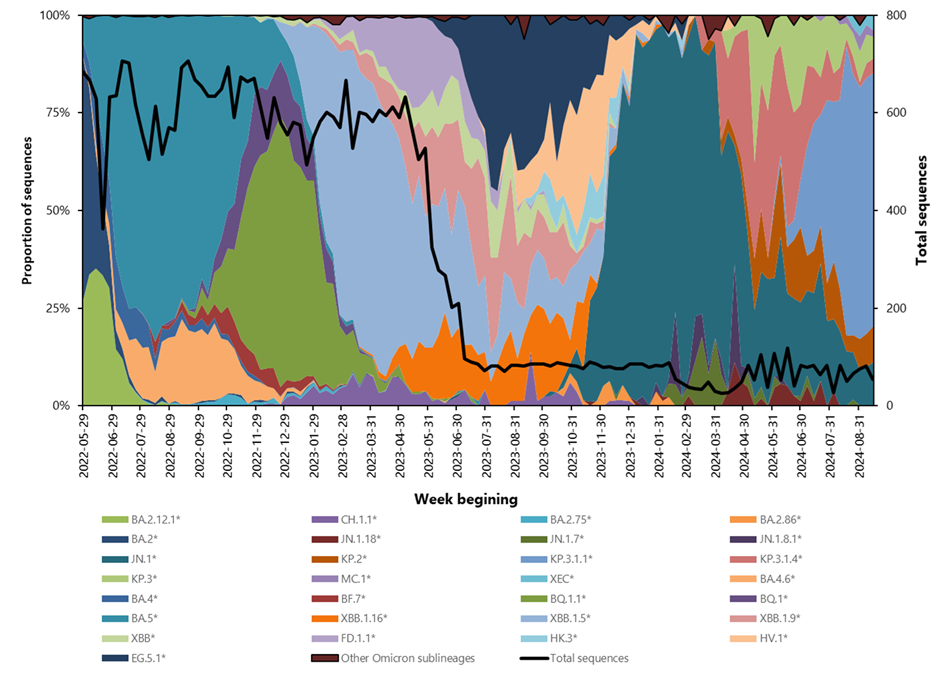
**

Figure caption: The asterix (*) refers to the specified Omicron sublineage and pooled descendent lineages, except those already shown in the figure. The figure was generated by Microsoft Excel (Version 2509 Build 16.0.19231.20246)

**Fig. S2**. Pairwise Wilcoxon test results of COVID-19 outbreak duration and size, and median weekly COVID-19 cases in long-term care facilities by surveillance period, region and bed capacity in Québec, Canada (epiweek 22-2022 to 38-2024)


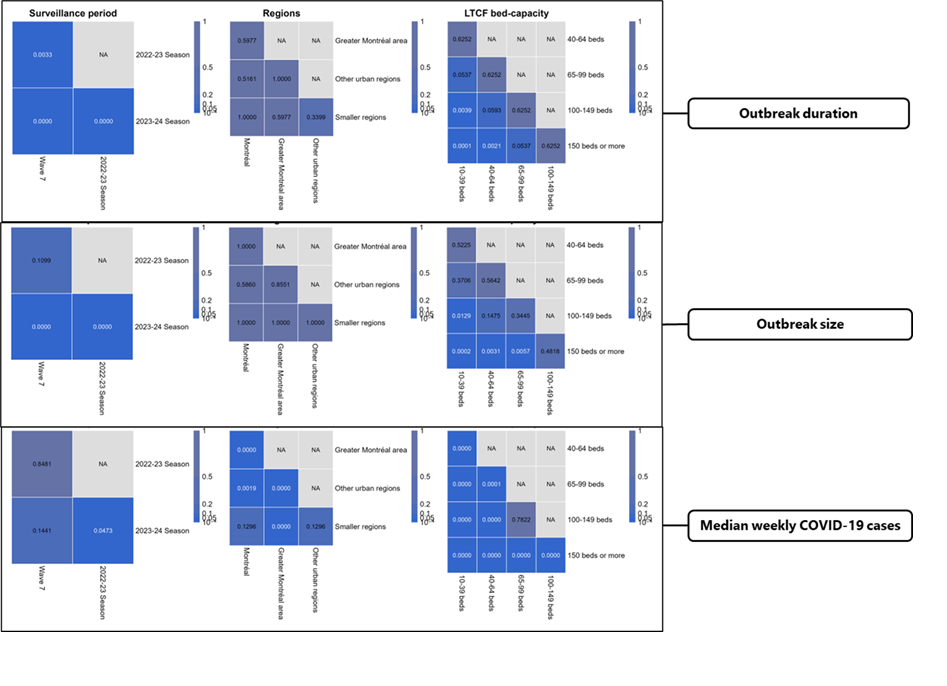


**Fig. S3**. Correlation between the number of COVID-19 outbreaks in long-term care facilities and weekly number of COVID-19 cases in the general population, Québec, Canada (epiweek 22-2022 to 38-2024)


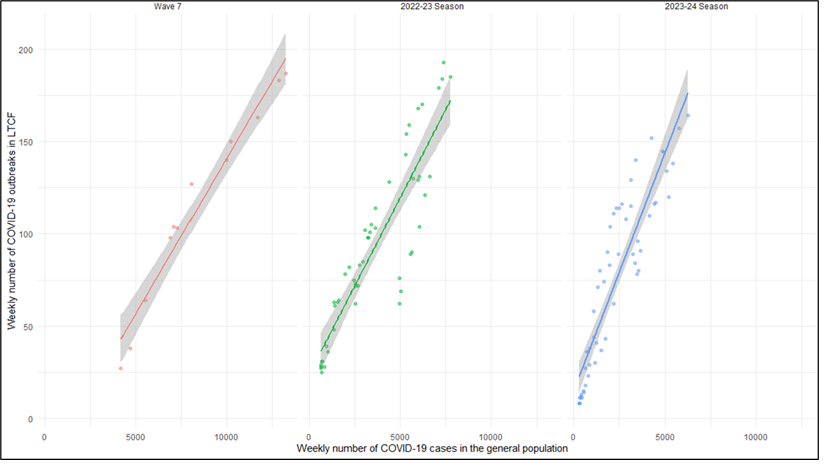


Figure caption: COVID-19 cases in the general population were lagged by one week compared to LTCF outbreaks. The period was defined based on the week of the outbreak onset. The shaded areas refer to the 95% confidence interval.

**Fig. S4**. Correlation between COVID-19 cases in long-term care facilities and weekly number of COVID-19 cases in the general population, Québec, Canada (epiweek 22-2022 to 38-2024)


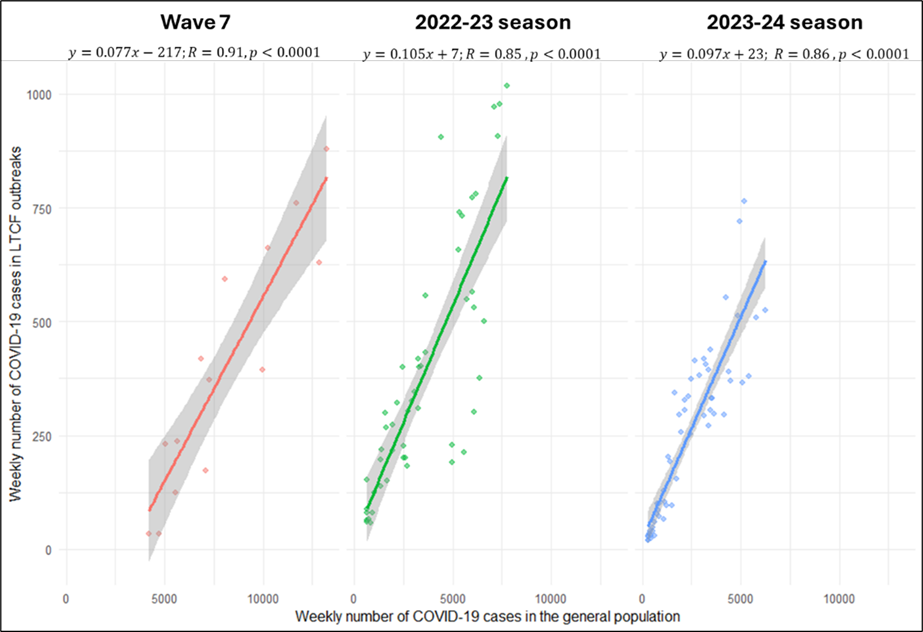


Figure caption: COVID-19 cases in the general population were lagged by two weeks compared to LTCF cases. The period was defined based on the week of the outbreak onset. The shaded areas refer to the 95% confidence interval.
